# The unique challenges of childhood-onset systemic lupus erythematosus and lupus nephritis patients: a proposed framework for an individualized transitional care plan

**DOI:** 10.1007/s00467-024-06654-5

**Published:** 2025-03-13

**Authors:** Thomas Renson, Liz Lightstone, Coziana Ciurtin, Claire Gaymer, Stephen D. Marks

**Affiliations:** 1 Pediatric Nephrology and Rheumatology, Department of Pediatrics and Internal Medicine, Ghent University Hospital, Ghent, Belgium; 2 European Reference Network for Immunodeficiency, Autoinflammatory, Autoimmune, and Pediatric Rheumatic Disease (ERN-RITA), Utrecht, Netherlands; 3 European Reference Network for Rare Kidney Diseases (ERKNet), Heidelberg, Germany; 4 Centre for Inflammatory Disease, Department of Immunology and Inflammation, Imperial College London, London, UK; 5 Imperial Lupus Centre, Hammersmith Hospital, Imperial College Healthcare NHS Trust, London, UK; 6 Department of Adolescent Rheumatology, University College London Hospitals NHS Trust, London, UK; 7 Centre for Adolescent Rheumatology, Division of Medicine, University College London, London, UK; 8 Department of Pediatric Nephrology, Great Ormond Street Hospital for Children, NHS Foundation Trust, London, UK; 9 NIHR Great Ormond Street Hospital Biomedical Research Centre, University College London Great Ormond Street Institute of Child Health, London, UK

**Keywords:** Childhood-onset systemic lupus erythematosus, Lupus nephritis, Adolescents and young adults

## Abstract

Childhood-onset systemic lupus erythematosus (cSLE) is a severe lifelong and life-threatening autoimmune disease with multi-organ involvement. Compared to those with adult-onset disease, cSLE patients have more aggressive disease with a higher prevalence of early lupus nephritis (LN) causing worse kidney and patient outcomes. The transfer of adolescent patients to adult healthcare poses several major challenges, from a disease as well as a psychosocial perspective. Transitional care even in tertiary centers can be heterogenous, suboptimal, and often even non-existent. In this comprehensive review of the literature, we synthesize the obstacles adolescents and young adults (AYA) with systemic lupus erythematosus (SLE) and LN face and how these challenges impact the transfer to adult health care. Finally, we propose a framework for a structured and individually modifiable transitional care plan, tailored to the unique needs of this population and taking into account their social and cultural background. This framework includes suggestions for the timing of the preparatory phase and the transfer itself, the composition of the transitional care team, increasing transition readiness and treatment adherence, and establishing a supportive network of peers. Efficient transitional care will optimize long-term patient outcomes.

**Graphical Abstract:**

**Figure:**
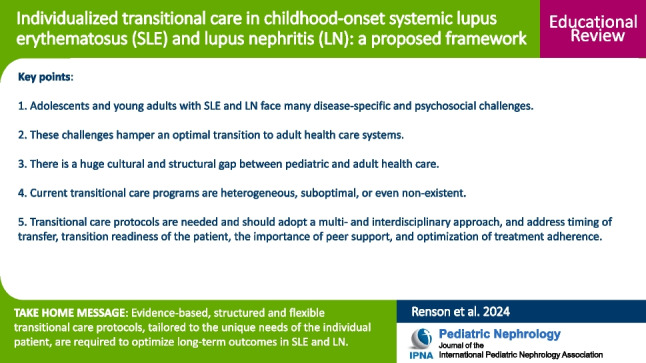
A higher resolution version of the Graphical abstract is available as Supplementary information

**Supplementary Information:**

The online version contains supplementary material available at 10.1007/s00467-024-06654-5.

## Introduction

SLE is a chronic auto-antibody-mediated disease with severe multi-organ system involvement. LN occurs in about 40% of SLE patients, mostly early in the disease course and often as the initial presentation [1, 2]. LN is a major cause of morbidity and mortality, with up to 10% of patients developing kidney failure, requiring dialysis and/or kidney transplantation [2]. Kidney involvement therefore carries an enormous burden on the quality of life of SLE patients.

Up to 20% of SLE patients present with cSLE, with a diagnosis before the age of 18 years [3]. However, some adults diagnosed with SLE have had symptoms before the age of 18 years for which they did not seek medical advice or were misdiagnosed [4]. cSLE patients generally demonstrate more severe disease with a higher incidence and more aggressive course of LN [5, 6]. cSLE was a strong predictor of mortality in the Lupus Outcomes Study, with a 5-year mortality rate of 12.5% [7]. cSLE patients are therefore a high-risk population with unique requirements.

Adolescents with cSLE face many obstacles and personal and psychological changes, which make them especially vulnerable. The transfer to adult health care is often challenging [8, 9] as only some centers have specific transition clinics. Moreover, the existing transition clinics have heterogeneous protocols, which challenges the comparison of data. There is a need for evidence-based guidelines for cSLE transition clinics, with a primary focus on LN, addressing the unique needs of this at-risk population. The objectives of this review are therefore to (1) synthesize the challenges faced by AYA with SLE and LN during the transition to adult health care and (2) propose a framework for an individualized transition trajectory which will optimize patient outcomes.

## Challenges of childhood-onset SLE and LN patients

### Disease-specific challenges

SLE patients with symptom onset in childhood are characterized by a more aggressive disease course with worse long-term outcomes compared with patients developing SLE later in life. Brunner et al. compared disease characteristics and patient outcomes of a cSLE inception cohort with an adult-onset SLE inception cohort [5]. Seventy-eight percent of the cSLE patients had LN, whereas about half of the adult patients had kidney involvement (*p* < 0.001). Adjusted mean SLE Disease Activity Index (SLEDAI) kidney scores were higher in cSLE patients (2.37 vs 0.82, *p* < 0.001). There was a higher requirement for treatment with corticosteroids (97% vs 72%, *p* < 0.001) and other immunosuppressive drugs (66% vs 37%, *p* < 0.001) in pediatric patients. Long-term accrual of organ damage is more prominent in cSLE patients as they present with higher Systemic Lupus International Collaborating Clinics/American College of Rheumatology Damage Index (SDI) scores compared to adult patients (1.70 vs 0.76, *p* = 0.008) which may be due to higher corticosteroid usage. Hersh et al. reported that cSLE patients have more kidney involvement and are more likely to need kidney replacement therapy with dialysis and/or kidney transplantation [6]. Despite improvements in immunosuppressive treatments, kidney outcomes remain disappointing with only 40 to 60% achieving complete kidney remission [10]. Groot et al. described a cohort of cSLE patients in which 9% of patients with LN proceeded to kidney transplantation at a median age of 24 years [11]. Flares of LN disease activity are frequently seen. Hui et al. demonstrated that one-third of patients with biopsy-proven International Society of Nephrology/Renal Pathology Society (ISN/RPS) Class III-V LN develop a flare after a mean post-induction time of 3.5 years [12]. Notwithstanding that long-term kidney outcomes of childhood-onset LN have improved over the past decades, LN relapse and failure to reach remission at 6 and 12 months remain significant predictors for advanced chronic kidney disease (CKD) and kidney failure [13–15]. Moreover, patients with childhood-onset LN are at higher risk of treatment-associated damage considering the higher requirement for corticosteroids and stronger non-selective or targeted immunosuppressive agents [5, 16].

### Psychosocial challenges

Maturation of the prefrontal cortex occurs in late adolescence and early adulthood, which impacts impulse control and rational decision-making at younger ages [17, 18]. As a result, AYA are less likely to optimally weigh risks and benefits, which can be detrimental in the context of a chronic health condition. AYA go through profound personal, social, and professional changes impacting body image and self-esteem with SLE patients facing multiple additional challenges. Diagnostic delay is substantial in SLE with time to cSLE diagnosis from symptom onset of 5 years in the Euro-lupus project, a large prospective cohort study comprising 1000 SLE patients [19]. The search for a diagnosis of SLE may be a true odyssey, causing frustration among AYA with new-onset lupus symptoms. Treatment adherence is of major concern in SLE with over half of patients being non-adherent to their medication [20–22]. Medication non-adherence is associated with higher damage and disease activity scores and higher utilization of acute care services [21–23]. AYA with SLE have a significant psychological burden. A systematic review by Quilter et al. reported depressive symptoms in 7 to 59% and anxiety symptoms in 34 to 37% of SLE patients [24]. These patients report feelings of being restricted from physical and social activities with issues of body image and self-esteem, leading to a sense of social isolation [25]. Moreover, they face a significant uncertainty about their future. SLE patients feel restricted in major life decisions, such as career choices and parenthood [26]. Therefore, AYA with SLE are especially vulnerable from a psychosocial perspective. Major challenges in these domains have an important impact on the overall health and long-term outcome of SLE patients. Research with the main objective of alleviating the psychological burden of SLE is prioritized by AYA patients [27] with adolescents remaining a forgotten group [28]. They are under-represented in research and feel a lack of recognition as a particular and distinct group.

### Challenges of minority populations

cSLE patients of non-Caucasian descent are a particularly vulnerable group. There is a higher prevalence of cSLE in patients of Asian, Hispanic, African American, and Native American descent [29]. Importantly, those ethnicities are also associated with worse outcomes. The prevalence of LN is also higher in several other groups, especially in women of African American and Asian descent [30]. African American and Hispanic patients with SLE are at higher risk for readmission within 30 days after hospitalization [31]. In the same study, LN was also a risk factor for re-hospitalization. There is a lack of qualitative age- and culturally appropriate patient education resources, which impacts patient independence and self-sufficiency [32]. Compared to juvenile idiopathic arthritis (JIA), SLE disproportionately affects ethnic minorities and socioeconomically disadvantaged AYA adding to their transition challenges and increasing the risk for reduced transition readiness [33–35].

## The difficult transition to the adult health care system

In many parts of the world, including Europe and North America, there is a large cultural and structural gap between pediatric and adult health care systems [36]. Pediatric health care is more paternalistically oriented and family-centered, whereas adult health care focuses on self-responsibility and self-accountability. Patients often feel isolated and insecure while bridging both health care systems. AYA with SLE feel uncomfortable with the prospect of new care providers [25]. Importantly, SLE patients report the transition process to adult health care as one of their main challenges.

The transition to adult health care systems is a vulnerable period for AYA with a chronic condition. In a retrospective cohort study by Hersh et al., almost 30% of patients with an inflammatory rheumatic condition were hospitalized for the management of active disease in the year prior to their transfer to adult care [37]. Over half of patients had active disease at the time of transfer. Nearly 30% of patients experienced a higher disease activity post-transfer. Hazel et al. reported that over 50% of JIA patients were lost to follow-up during transition to adult health care [38]. Felsenstein et al. demonstrated transition difficulties in over half of cSLE patients [9]. Post-transition, 90% of patients described recent symptoms of active SLE. Forty-one percent of patients even developed new kidney manifestations. Son et al. assessed 50 cSLE patients after transfer to an adult rheumatologist [8]. SLICC scores were significantly higher post-transfer (0.46 ± 0.84 vs 0.78 ± 1.25, *p* = 0.01). A quarter of patients suffered from feelings of depression and/or anxiety, whereas this was only the case for 10% before transfer (*p* = 0.02). The mean time between last visit with a pediatric rheumatologist and the first encounter with an adult rheumatologist was nearly 9 months, with almost 75% experiencing a gap in their continuity of care (i.e., one or more missed appointments). In a study involving 141 pediatric patients with a rheumatic condition, the mean time between last pediatric and first adult health care visit was more than 7 months [39]. Diagnosis of a connective tissue disease (including SLE) was a risk factor for hospitalization and emergency department visits. In a recent study, Chang et al. evaluated patterns of health care use by AYA with SLE during the transition period [40]. Over one quarter of patients failed to consult an adult rheumatologist or nephrologist within 12 months of their last pediatric visit. Furthermore, these patients demonstrated worse medication adherence. Continuity of care may even be more impeded if AYA move to a different city, e.g., to attend college. Joint pediatric-adult clinics will consequently not be feasible. In this context, multiple virtual meetings between the different care teams and strict clinical follow-up may smoothen this challenging transition period. The above raised issues and cited studies underscore the requirement for a culture change in the management of AYA with chronic health conditions transferring to adult health care.

Finally, transition trajectories all over the world are heterogeneous. There is a lack of a widely adoptable set of recommendations. Results of a 2022 ERN-RITA survey demonstrated that 75% of the primary immunodeficiencies and autoinflammatory diseases centers have a defined transition process, albeit many lack national disease-specific transition guidelines [41]. It is therefore challenging to define a successful or optimal transition to adult health care. There is no single outcome measure assessing transition success, which hampers comparative studies [42]. Practical guidelines in the assessment of transition effectiveness are warranted to accommodate future research.

## Framework for the transition of childhood-onset SLE/LN patients

Transitional care was defined by the Society for Adolescent Medicine as the “purposeful, planned movement of AYA with chronic physical and medical conditions from child-centered to adult-oriented health care systems” [43]. The actual transfer from pediatric to adult health care should be embedded in an overarching transition process. This process may comprise three subsequent phases: a preparatory pre-transfer phase, the actual transfer itself, and a post-transfer phase. Moreover, every center should have a written transitional care policy or protocol available [42]. There is a need for structured, standardized, and individually modifiable (i.e., tailored to the unique needs of individual patients) transition programs involving multi-disciplinary care. Importantly, specific cSLE and LN transition programs may not be feasible in many centers. In this setting, transition can be organized within overarching autoimmune/rheumatic disease, CKD or (renal) vasculitis transition clinics, or general transition clinics aimed at AYA with chronic disease. cSLE patients were included in the study by Overbury et al., reporting on the implementation of the Adult Center for Childhood Onset Rheumatic Disease transition clinic. However, whether outcomes of the cSLE patients participating in this transition program improved was not specifically reported. The European Alliance of Associations for Rheumatology and the Pediatric Rheumatology European Society published a well-defined framework for youth with childhood-onset rheumatic disease. Notwithstanding that such guidelines function as a good starting point for the transition of cSLE and LN patients, they lack the incorporation of disease-specific challenges, such as the need for both rheumatology and nephrology care services. Moreover, they often miss the CKD vantage point, including the potential need for kidney replacement therapy. In this educational review, we present a framework for the transition of cSLE and LN patients in particular (Table 1).

**Table 1:** Proposed framework for the transition of cSLE and LN patients

*General*	• Establish a local individually-modifiable transition policy • Three transition phases: pre-transfer, transfer, post-transfer
*Timing*	• Initiate transition process early: - At time of diagnosis - In early adolescence (age 11–13) • Timing of actual transfer is variable: - Transition readiness - Stable period, quiescent disease • In general: initiate transition early, actual transfer may be better late
*Assess and optimize transition readiness*	• Patient development • Self-management skills • Self-advocacy • Gradually implement shared-decision making • Personalize patient education • Set and manage patient expectations
*Multi- and inter-disciplinary approach*	• Composition of the medical and allied health care team: - Pediatric rheumatologist/nephrologist - Adult rheumatologist/nephrologist - Specialist nurse, transition coordinator - Clinical psychologist - Involve general practitioner • Organize joint pediatric-adult transition clinic appointments • Treatment: shared decision between all parties • Refrain from treatment withdrawal during transition
*Enhance peer support*	• Organize peer support events • Acknowledge the specific needs of minority populations
*Optimize treatment adherence*	• Emphasize the importance of therapy compliance • Push notifications on mobile phone • Medication calendars • Pill organizers, blister packages • Consider therapeutic drug monitoring

### Timing

The transition process should be initiated early, ideally in early adolescence (age 11 to 13 years) or even at time of diagnosis [42, 44]. The timing of the actual transfer to adult health care services is less evident, as this is highly dependent on the transition readiness of patients and their families as well as hospital policies. Health care transition should be developmentally appropriate rather than depending on patient age [45]. A transitional care model with emphasis on augmenting skills in self-management and self-advocacy is therefore required [28]. Patients should be able to effectively and independently navigate within the existing health care systems at the time of transfer to adult care [28]. In general, the actual transfer should be implemented during a stable period, from a disease perspective (i.e., inactive disease) as well as a personal psychosocial perspective [44] and usually between patients’ 16th and 18th birthdays depending on local healthcare guidelines. Foster et al. assessed the impact of transfer timing on kidney allograft survival in a large study involving 440 kidney transplant recipients [46]. It was noted that those patients that were transferred before the age of 21 years had a 58% higher risk of kidney allograft failure compared to those patients transferred late (95% confidence interval 7–134%, *p* = 0.02). There is evidence to suggest that transitional care processes should be initiated in early adolescence with delayed transfer at a later date.

### Transition readiness

Self-management is an important aspect in the transition readiness of AYA with chronic conditions. Pediatric health care is paternalistically oriented from a cultural point-of-view. Young patients should be progressively involved in the management of their disease with shared decision-making gradually implemented. In this regard, repeated patient education on their disease and its optimal management is fundamental. Patient education should be personalized, developmentally and culturally appropriate, and offered in several formats, including printed text, webpages, and educational videos. Setting the expectations of transitional care with the patient and their family before transfer is essential [47, 48]. An individualized transitional care plan can subsequently be composed. Moreover, transition readiness should be regularly assessed using checklists documented in the patient’s notes or electronic patient records [47].

### A multi- and inter-disciplinary approach

The transitional care team of patients with SLE and LN should include both medical and allied health care specialties with medical physicians including pediatric nephrologists and rheumatologists and adult nephrologists and rheumatologists. Meeting the respective adult care providers before transfer is essential to optimize transition outcomes for patients [40, 49]. Pediatric and adult physicians may provide at least one joint clinic visit to smooth transition and coordinate treatment plans. Clinic appointments should be scheduled in a timely manner to minimize the risk of disease flares and drop-outs during transition. In case of missed appointments, patients should be actively encouraged to schedule a new date. A specialist nurse may act as a transition coordinator and provide continuity by joining both the pediatric and adult care team in clinic. The specialist nurse may also aid in discussing issues specifically related to AYA, such as the use of contraceptive measures considering the teratogenicity of several medications used in the treatment of SLE and LN. As continuity during the transition process should not be neglected, the patient’s general practitioner or primary care provider can be actively involved [50]. Moreover, it is advisable that the pediatric care team stays accessible for the patient between the last joint pediatric-adult clinic and the first adult clinic appointment, which should be scheduled within a time period of 3 months. Finally, clinic visits with a psychologist specialized in AYA with CKD may be of added value for AYA with LN. Patients appreciate the involvement of a highly coordinated team offering comprehensive care [27]. A balanced multi-disciplinary care team can provide AYA the required guidance while they are navigating this difficult transition process.

The optimal treatment plan should be agreed upon by both the pediatric physician and the adult nephrologist/rheumatologist, ideally in a joint clinic, and should involve shared decision-making by all parties (pediatrician, adult physician, and patient). It is important to note that new therapeutic agents may become accessible once a patient is 18 years of age, as per reimbursement conditions. Nonetheless, switching to different treatment agents while a patient is in remission may lead to adverse outcomes. Considering the important gaps in the continuity of care during the transition period, an in-hospital intravenous treatment such as rituximab may be considered to optimize treatment adherence. However, the efficacy of rituximab in SLE and LN remains the subject of debate. Although multiple reports have suggested a benefit for its use [51–54], the LUNAR study, a randomized, double-blind, placebo-controlled trial assessing the efficacy of rituximab in LN patients, failed to reach its primary endpoint, i.e., superior response rates with rituximab compared to placebo [55]. Obinutuzumab might lead to deeper B cell depletion and higher complete kidney response rates [56]. Finally, the optimal treatment duration in childhood-onset LN remains elusive. Current guidelines advocate for a minimum treatment duration of 3 years [57]. Considering the risk of flares of disease activity and gaps in the continuity of care, it seems advisable to wait with treatment withdrawal until transition is complete and successful.

### The importance of peer support

AYA with SLE and LN often feel restricted on both a physical and social level. Considering the heterogeneity and intangibility of the disease, they often have a sense of alienation. Living with CKD may augment these negative emotions. Organized peer support events, such as lupus recreational camps, may therefore be extremely valuable. These events have the potential to increase social connection between patients, medication adherence, patient education, and feelings of disease acceptance, independence, and self-efficacy [58, 59]. AYA-focused lupus camps can provide feedback on unmet needs of transitional care to guide future frameworks and should therefore be offered to SLE and LN patients during their transition process. These events seem particularly beneficial for minority populations who may have additional requirements.

### Treatment adherence

Medication adherence is a major challenge in SLE patients, considering the polypharmacy and associated side-effects of many drugs. Non-adherence is common and is associated with worse SLE outcomes [20–23]. The importance of medication adherence should be emphasized during transition, with consideration of intravenous medications in selected patients. Several tools can aid patients with their treatment compliance, including mobile phone applications sending daily push notifications, medication calendars, and pill organizers. Physicians can use therapeutic drug monitoring to assess treatment adherence (including tacrolimus and hydroxychloroquine levels). Pharmacies can offer assistance by providing pre-arranged blister packages. Support by peers is also of added value in enhancing treatment adherence.

## Conclusion

AYA with SLE, especially those patients with LN, are a vulnerable population. cSLE patients have a more aggressive disease course, higher prevalence of kidney involvement, more damage accrual, and worse kidney and patient outcomes. Moreover, both their current stage of life and living with a chronic and severe disease cause significant psychosocial obstacles, such as negative feelings of low self-esteem and body image, anxiety, depression, social isolation, and risk of treatment non-adherence. AYA of non-Caucasian descent are an especially vulnerable subgroup. All these aspects put AYA with SLE and LN at risk for a challenging transition period to adult health care with high risk of gaps in the continuity of care and lupus flares.

A structured and individually modifiable transition protocol for an optimal transition of AYA with SLE and LN is required. This plan should be tailored to the specific and unique needs of individual patients, taking into account their social and cultural background. Here, we present a framework for such a protocol. The preparatory pre-transfer stage of the transition process should start in early adolescence from 11 to 13 years. The timing of the actual transfer to adult health care is highly dependent on the transition readiness of the patient and their family, as well as hospital policies, but might be more efficient when executed later rather than earlier. Transition readiness should be enhanced during the pre-transfer phase by progressively augmenting patients’ self-responsibility and self-accountability and providing patient education on multiple occasions. Establishing a peer support network by organizing patient events such as lupus camps may aid in increasing transition readiness and treatment adherence. Importantly, the transitional care team should be multi-disciplinary and well-balanced, comprising at a minimum a pediatrician (pediatric nephrologist and/or rheumatologist), adult nephrologist and/or rheumatologist, a specialist nurse who may act as a transition coordinator, and a psychologist with expertise in AYA with a chronic (kidney) condition. Furthermore, the general practitioner or primary care provider should be involved to increase the continuity of care. A structured and well-organized transition clinic will optimize outcomes of AYA with SLE and LN and is therefore needed in each tertiary care center.

## Key summary points

1. Adolescents and young adults with systemic lupus erythematosus and lupus nephritis, particularly those of minority descent, face many disease-specific and psychosocial challenges, which hamper an optimal transition to adult health care systems.
2. Currently, there is a huge cultural and structural gap between pediatric and adult health care with heterogeneous, suboptimal, and even non-existent transitional care programs in many tertiary nephrology and rheumatology centers.
3. Evidence-based, structured and flexible transitional care protocols, tailored to the unique needs of the patient and taking into account their social and cultural background, are required to optimize long-term outcomes.

## Multiple choice questions

Answers appear following the references.

1. Which statement on systemic lupus erythematosus and lupus nephritis is true?
  A) Lupus nephritis occurs typically late in the disease course of childhood-onset systemic lupus erythematosus patients and is therefore often a major driver of disease activity during the transition period
  B) Up to one third of SLE patients are diagnosed before the age of 18 years
  C) Lupus nephritis is more prevalent and tends to behave more aggressively in children and adolescents compared to adult patients
  D) Up to 75% of lupus nephritis patients achieve complete kidney remission due to novel immunosuppressive agents and treatment regimens
2. Which research objectives are prioritized by adolescents and young adults with systemic lupus erythematosus?
  A) Novel targeted treatment options
  B) Alleviating the psychological burden
  C) Improving kidney outcomes
  D) Optimizing the transition to adult health care systems
3. Which statement on minority populations with systemic lupus erythematosus is false?
  A) The prevalence of systemic lupus erythematosus is the highest in people of Caucasian descent, but outcomes are worse in Asian, African American, Hispanic, and Native American patients
  B) The prevalence of lupus nephritis is particularly high in women of African American and Asian descent
  C) African American patients, Hispanic patients, and lupus nephritis patients are prone to readmission after hospitalization
  D) There is a lack of culturally appropriate patient education on systemic lupus erythematosus
4. Which statement on the transition to adult health care systems is true?
  A) The gap between pediatric and adult health care is bridged in many parts of the world
  B) Although loss to follow-up is a major issue during the transition period, most adolescents and young adults with systemic lupus erythematosus have quiescent disease
  C) Seventy-five percent of patients with systemic lupus erythematosus in transition experience a gap of care
  D) There are several validated outcome measures assessing transition effectiveness
5. Which statement on the timing of transition to adult health care is false?
  A) The transition period may already be initiated at the time of diagnosis
  B) The timing of transition depends particularly on the transition readiness of the patient’s parents
  C) Transitional care should be developmentally appropriate rather than age-based
  D) Adolescent and young adult patients should be able to independently navigate health care systems before transfer to adult care can occur

## Supplementary Information

Below is the link to the electronic supplementary material.

Graphical abstract (PPTX 61 KB)
