# Supplementary material for: The unique challenges of childhood-onset systemic lupus erythematosus and lupus nephritis patients: a proposed framework for an individualized transitional care plan
Source: Pediatr Nephrol. 2025 Mar 13;40(10):3045–53. doi: 10.1007/s00467-024-06654-5 (PMC12401761; doi:10.1007/s00467-024-06654-5)
Supplement: Supplementary file 1 — Graphical abstract (PPTX 61 KB) [file 467_2024_6654_MOESM1_ESM.pptx]

## Slide 1
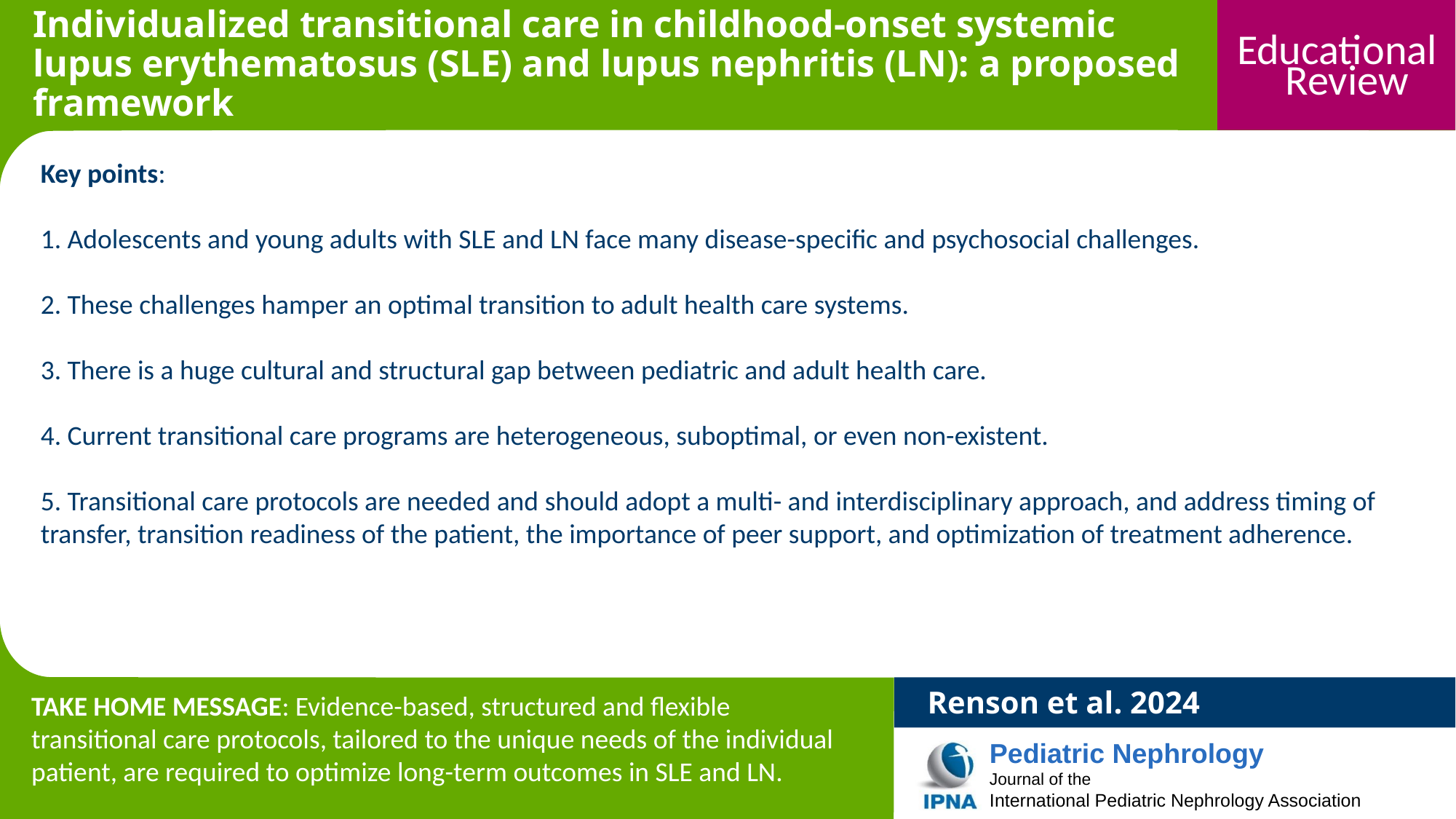

Individualized transitional care in childhood-onset systemic lupus erythematosus (SLE) and lupus nephritis (LN): a proposed framework
Key points:
1. Adolescents and young adults with SLE and LN face many disease-specific and psychosocial challenges.
2. These challenges hamper an optimal transition to adult health care systems.
3. There is a huge cultural and structural gap between pediatric and adult health care.
4. Current transitional care programs are heterogeneous, suboptimal, or even non-existent.
5. Transitional care protocols are needed and should adopt a multi- and interdisciplinary approach, and address timing of transfer, transition readiness of the patient, the importance of peer support, and optimization of treatment adherence.
Renson et al. 2024
TAKE HOME MESSAGE: Evidence-based, structured and flexible transitional care protocols, tailored to the unique needs of the individual patient, are required to optimize long-term outcomes in SLE and LN.
